# Hospital Committees and Hospital Secretaries

**Published:** 1896-01-04

**Authors:** 


### The Institutional Workshop.

### HOSPITAL COMMITTEES AND HOSPITAL SECRETARIES.

The proceedings at the special meeting of the governors at the Great Northern Central Hospital, Islington, on the 30th ult. were instructive in several ways. The voting illustrated the large amount of influence which is held by the honorary medical staff of certain institutions in London. At the Great Northern Central Hospital a system has arisen by which members of the medical staff and members of the committee not only become governors themselves, but make their wives governors also, so that collectively the staff and the committee have a solid phalanx of probably 100 votes always ready to vote together and to maintain the *status quo*. One result of this method of procedure was strikingly illustrated at the meeting which was summoned on the 30th ultimo, "to consider the question of secret commission alleged to have been paid to the late secretary and the concealment of these payments in the balance sheet issued by the committee." Mr. Bax, as will be seen by the account of the meeting published elsewhere, moved a strong vote of censure, which was duly seconded. Two independent governors moved an amendment to the resolution of condemnation which was so worded as to give the committee credit for what they had done, whilst it recorded a mild censure on some of their proceedings which no one—not even a committeeman—could defend as justifiable. At the request of the supporters of the amendment, Mr. Bax consented to withdraw his resolution, and the amendment was accepted as the substantive motion. The vote of censure thus being disposed of, to the surprise and regret of many of the best friends of the hospital, members of the committee rose one after another and urged, and ultimately carried by their own votes and the votes of their wives, the omission of a material word from the amendment which had been accepted in good faith by all the independent governors. The chairman, Mr. Murdoch, whose conduct in the chair was in pleasing contrast to his attitude on a former occasion, behaved very fairly, and did his best to discourage the omission of this word "justly," as he wisely foresaw that serious consequences might follow from such a demonstration of the solidarity of the committee, and their determination to set aside the views and wishes of all the independent governors present. Such a policy may be magnificent, but it is calculated to prove highly dangerous to the progress of a great charity, and if it is continued the interests of the Great Northern Central Hospital must be injured.

A curious phase of the discussion was the contention of some members of the committee that, as the whole committee is elected every year—a most pernicious system by-the-bye—the present committee has no responsibility for the proceedings of any former committee, although the *personnel* is practically identical. On this astounding assumption certain members of the committee maintained that the committee of the Great Northern Central Hospital, as at present constituted, is only responsible for its actions during its year of office, at the expiration of which time, on the re-election of its members, the governors have no power to secure redress or to apportion censure. It follows, as one governor present wisely pointed out, the present system of electing the committee is dangerous to the well-being of the hospital, and the laws ought to be altered so as to provide that one-third of the committee should retire at the end of each year, none of the retiring members being eligible for re-election for twelve months. We hope that a deputation of the governors may be organised to wait upon the committee with a view to secure this amendment of the laws before the next annual meeting.

Incidentally the question of the remuneration of hospital secretaries came under discussion. Some governors seem to think that a hospital secretary is a gentleman of so little importance that a merely nominal salary should be paid to him. As a matter of fact, there is no office connected with the business of charity which demands higher qualities from the holder than that of secretary to a great voluntary hospital. Experience has proved that it is the truest economy to pay a relatively high salary to such an officer, providing he is really competent for the varied duties of the office. Small and inadequate salaries will only attract the services of small and inexperienced men, to the great injury of a hospital, both to its prestige and financial strength. Whatever may be said against Mr. Grant, the late secretary of the Great Northern Central Hospital, as the Chairman proved in the course of his speech, he was undoubtedly a man of considerable business ability, who worked zealously and well for the interests of his hospital, and rendered real service to the institution at a most critical period of its history. We are strongly of opinion that it would be a most useful step for *The Hospitals' Association* to initiate a discussion on the duties and emoluments of hospital secretaries, because the time has come when the hospital governors and committees ought to be aroused to the importance of securing the best men for this responsible position, which can only be done by placing their salaries on a footing commensurate with the market value of the services of the trained expert, who alone can adequately finance a great voluntary hospital under present conditions. Several hospital secretaries, excellent and industrious men, are at present underpaid, and the sooner this injustice to every such institution and its officers is removed the better.

